# Potential Savings of Harmonising Hospital and Community Formularies for Chronic Disease Medications Initiated in Hospital

**DOI:** 10.1371/journal.pone.0039737

**Published:** 2012-06-26

**Authors:** Lauren Lapointe-Shaw, Hadas D. Fischer, Alice Newman, Ava John-Baptiste, Geoffrey M. Anderson, Paula A. Rochon, Chaim M. Bell

**Affiliations:** 1 Department of Medicine, University of Toronto, Toronto, Ontario; 2 Institute for Clinical Evaluative Sciences, Toronto, Ontario; 3 Women’s College Research Institute, Women’s College Hospital, Toronto, Ontario; 4 Institute of Health Policy, Management and Evaluation, University of Toronto, Toronto, Ontario; 5 Li Ka Shing Knowledge Institute, St. Michael’s Hospital, Toronto, Ontario; Yale University School of Medicine, United States of America

## Abstract

**Background:**

Hospitals in Canada manage their formularies independently, yet many inpatients are discharged on medications which will be purchased through publicly-funded programs. We sought to determine how much public money could be saved on chronic medications if hospitals promoted the initiation of agents with the lowest outpatient formulary prices.

**Methods:**

We used administrative databases for the province of Ontario to identify patients initiated on a proton pump inhibitor (PPI), angiotensin-converting enzyme (ACE) inhibitor or angiotensin receptor blocker (ARB) following hospital admission from April 1^st^ 2008-March 31^st^ 2009. We assessed the cost to the Ontario Drug Benefit Program (ODB) over the year following initiation and determined the cost savings if prescriptions were substituted with the least expensive agent in each class.

**Results:**

The cost for filling all PPI, ACE inhibitor and ARB prescriptions was $ 2.48 million, $968 thousand and $325 thousand respectively. Substituting the least expensive agent could have saved $1.16 million (47%) for PPIs, $162 thousand (17%) for ACE inhibitors and $14 thousand (4%) for ARBs over the year following discharge.

**Interpretation:**

In a setting where outpatient prescriptions are publicly funded, harmonising outpatient formularies with inpatient therapeutic substitution resulted in modest cost savings and may be one way to control rising pharmaceutical costs.

## Introduction

Annual healthcare expenditures in Canada are on a steep upward climb, reaching $ 191 billion in 2010 [1]. Medications represent an increasing share of costs, currently about 16%. A few classes of drugs account for the bulk of expenses [2]. For instance, the annual cost of angiotensin-converting enzyme (ACE) inhibitors in Canada doubled over the previous ten years to reach $ 956 million in 2006 [3]. In this climate, healthcare managers have turned to therapeutic substitution and reference-based pricing in order to contain costs [4], [5]. While therapeutic substitution targets agent selection by reducing formulary options, reference-based pricing limits prescription reimbursement to the cost of the least expensive equivalent drug. Canada’s provincial premiers have even spoken of creating a national pharmaceutical purchasing agency in order to take advantage of economies of scale [6].

Publicly-funded programs absorb the cost of a large proportion of outpatient drug expenditures [2]. Past studies have shown that what is prescribed in hospital drives ongoing prescription in the community[7]–[10]. Hospitals depend on public funds yet negotiate drug prices directly with suppliers or through group-purchasing organisations[11]–[13]. In either case, medication prices negotiated by hospitals may not match those of the public-payer’s outpatient formulary. Suppliers can offer hospitals discounts on proprietary drugs in order to secure a client-base. In-hospital therapeutic substitution strategies may steer patients toward agents that are inexpensive for the hospital, yet more expensive for public drug programs once a patient is discharged home. Such agents become “loss leaders”; their favourable pricing in one instance is used to generate profits later on. This can have a perverse effect on long-term drug costs once in the community.

Even small differences in drug acquisition costs can be amplified over time because of the long-term nature of chronic disease therapy. A harmonisation strategy would employ therapeutic substitution in order to direct inpatients toward the least expensive outpatient agents. We sought to determine how much savings could be achieved on selected chronic medications if hospitals initiated agents with the lowest outpatient formulary prices.

## Methods

### Overview

We used population-based administrative data covering all Ontario residents over the age of 65. Patients were selected if they were initiated on an ACE inhibitor, angiotensin receptor blocker (ARB), or proton pump inhibitor (PPI) following hospitalization. We assessed the cost to the Ontario Drug Benefit Program (ODB) over the year following initiation. We then compared this cost to the equivalent cost if all medications were substituted with the least expensive agent in that category. Our primary outcome was cost savings if this least expensive agent was used. This study was approved by the research ethics board of Sunnybrook Health Sciences Centre in Toronto, Canada.

### Participants

This study made use of the multiple linked healthcare databases available through the Institute for Clinical Evaluative Sciences (ICES). These data were linked via encrypted unique patient identifiers. Consent for participation was not obtained from individual patients. This administrative data is collected by governmental agencies and shared with ICES for research purposes. Data are protected and pooled in order to prevent individuals from being identifiable. We combined information from the Ontario Drug Benefit (ODB) database, the Canadian Institute for Health Information Discharge Abstract Database (CIHI-DAD), the Ontario Health Insurance Plan (OHIP) physician billing services database and the Registered Persons Database (RPDP). The comprehensive nature of health insurance coverage in Ontario allows consideration of these analyses as population-based. Data extracted using our databases has been validated in previous studies [14], [15].

Our cohort included all individuals aged 66 and over who filled a new prescription for an ACE inhibitor, ARB or PPI within 7 days following hospital discharge between April 1^st^ 2008 and March 31^st^ 2009. Prescriptions filled within 7 days of hospital discharge have been used previously as a surrogate marker of discharge prescription [8]. These drug classes were analysed in three separate cohorts.

ACE inhibitor, ARB and PPI medication classes were selected for two reasons. These medications are typically used to treat common chronic medical conditions frequently discovered during hospitalization[16]–[19]. Furthermore, the classes contain multiple agents often considered to be of similar efficacy and side effect profile. These are also medication classes commonly targeted by therapeutic substitution policies [20], [21].

Information concerning dispensed medications was identified using ODB claims. These data provide medication information using a drug identification number (DIN) specific to each medication and dose, as well as the quantity, days’ supply and the total prescription cost. These data have proven reliable when compared to pharmacy prescription audit [15]. The ODB program provides outpatient medication coverage to patients over age 65. As part of this program, patients make a fixed per-prescription co-payment and high-income patients also pay a $100 annual deductible [22].

### Inclusions and Exclusions

In each of the three medication categories, patients were excluded if the prescription was for a combination agent or if they had filled a prescription for a medication within the same class in the year prior to admission. Patients were excluded from the ACE inhibitor group if they received captopril because it requires multiple daily dosing and as such is markedly different from the other ACE inhibitors. Patients were excluded from the PPI group if they also received clopidogrel because of the possibility that drug interactions were guiding medication selection [23]–[26].

In order to capture the effect of one admission, patients transferred to another institution were excluded. Patients with an index admission of more than 30 days were also excluded as these patients may differ in their medication usage. For instance, a longer admission may increase likelihood of multiple agents being tried within a category.

### Patient Characteristics

We collected information on age, sex and socioeconomic status (low versus high income based on co-payment to ODB). We also recorded Charlson comorbidity index [27], type of hospital setting (urban, academic) and length of hospital stay. The number of patients in long term care before and after index admission was also recorded.

### Outcome

The primary outcome was cost of therapy over the year following hospital discharge. Net savings to the ODB if the discharge medication was substituted with a less expensive agent from the same class were then calculated. A secondary outcome measurement was the cost of inpatient coverage of medication costs based on ODB prices.

### Calculation of Potential Savings

Real cost of therapy was obtained directly from ODB claims. This was compared to the theoretical cost of therapy using an equivalent dose of the least expensive agent in that class.

The least expensive agent within each class was identified using ODB formulary prices in effect from April 1^st^ 2008 to March 31st 2010 [28]. Within each class, prices were compared using equivalent doses. The theoretical cost of therapy with this agent was then determined by multiplying the total number of pills dispensed by the unit cost of the inexpensive agent as listed on the formulary. Where the unit cost varied over our time period, we used the price in effect for the greatest proportion.

Equivalent dose selection was based on the World Health Organisation’s Defined Daily Doses [29]. Doses were modified to reflect available formulations (see Tables S1, S2 and S3). When an equivalent dose formulation was unavailable (eg: enalapril 2.5 mg in ramipril equivalents) then the closest dose match was used. For a low dose, this was the next highest dose; for a high dose, two tabs of the highest dose were used.

A secondary analysis calculated the maximum cost to the provincial drug plan of covering in-hospital medications. This was obtained by multiplying total length of stay by the daily cost of the first discharge prescription agent. This assumes the discharge agent was administered every day of the hospitalisation period. The daily cost with the discharge agent was chosen rather than a hypothetical daily cost with the least expensive agent in order to make this estimate more realistic.

## Results

The mean age was similar in the three groups, 78–79 years (Table 1). Most patients had co-morbid conditions as evidenced by Charlson score, and median length of stay was 6 days. Of the patients alive at one year following discharge, 44% were still filling prescriptions for the discharge PPI, and 59% were filling prescriptions for the discharge ACE inhibitor and ARB agents. For the three groups in the year following discharge, hospital readmission rates were 40–43% and 14–20% of patients died.

**Table 1:** Patient characteristics and prescription outcomes for PPI, ACE inhibitor and ARB groups.

	PPI*	ACE inhibitor †	ARB‡
Total in Group, n	7 892	6 802	963
Age at Index Date, Median (IQR), y	78 (72–84)	78 (72–84)	79 (73–84)
Female sex, no. (%)	4 117 (52.2)	3 463 (50.9)	563 (58.5)
Low-income status, No. (%)	1 913 (24.2)	1546 (22.7)	252 (26.2)
Charlson index, n (%)§			
0	1 927 (24.4)	1172 (17.2)	230 (23.9)
1	2 079 (26.3)	2 511 (36.9)	274 (28.5)
≥2	3 886 (49.2)	3 119 (45.9)	459 (47.7)
Urban Hospital Setting n (%)	7 251 (91.9)	6 247 (91.8)	869 (90.3)
Teaching Hospital n (%)	1 710 (21.7)	1 774 (26.1)	147 (15.3)
Median length of Stay During Index n (IQR)	6(3–11)	6 (3–10)	6 (3–10)
Admitted from Long-term care, n (%)	621 (7.9)	402 (5.9)	53 (5.5)
Discharged to Long-term care, n (%)	810 (10.3)	514 (7.6)	78 (8.1)
**Follow up within 1 year of discharge:**	**PPI** *	**ACE inhibitor** †	**ARB** ‡
Days survived, mean ± SD	317.69±105.63	334.47±85.86	332.69±87.83
Died within 365 days of Discharge, n (%)	1 580 (20.0)	958 (14.1)	148 (15.4)
Patients getting 2+ different drugs in same class, n (%)	1 146 (14.5)	239 (3.5)	39 (4.0)
Patients on first prescribed agent at one year following discharge, n (% of alive)	2 806 (44.5)	3464 (59.3)	485 (59.5)
Patients on any agent in class at one year following discharge, n (% of alive)	3 373 (53.4)	3599 (61.6)	509 (62.5)
Patients readmitted to hospital, n (%)	3 420 (43.3)	2 748 (40.4)	414 (43.0)
Total days in Hospital, mean ± SD	19.51±27.52	17.19±23.97	20.30±36.76

* Proton pump inhibitor: omeprazole, pantoprazole, lansoprazole or rabeprazole.

† Angiotensin-converting enzyme inhibitor: ramipril, enalapril (maleate and sodium), quinapril, fosinopril, lisinopril, benazepril, perindopril, cilazapril or trandolapril.

‡ Angiotensin receptor blocker: losartan, candesartan, irbesartan, valsartan, telmisartan or eprosartan.

§ As defined by Charlson comorbidity index [26].

### Proton Pump Inhibitors

**Figure 1:**
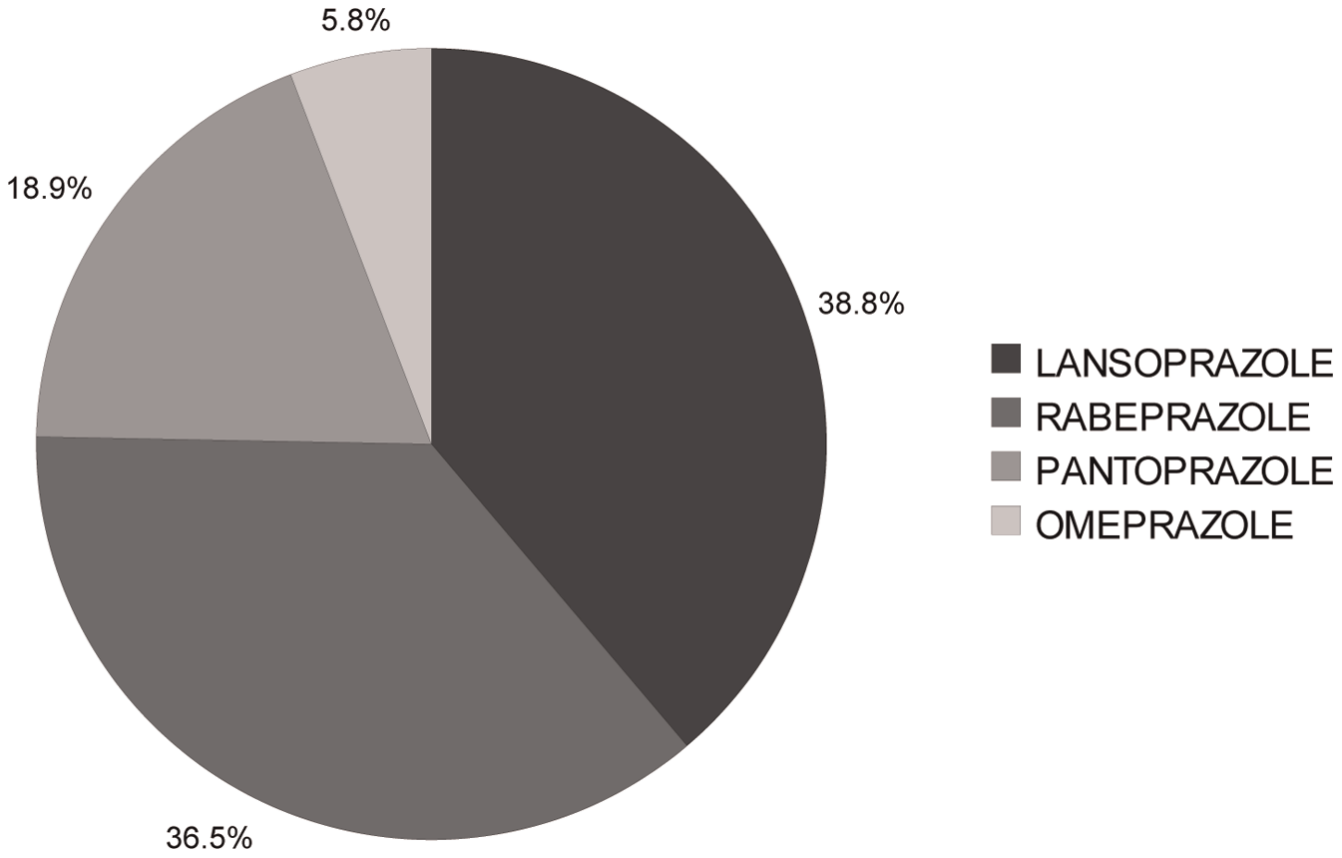
First Proton Pump Inhibitor Prescription Filled After Hospital Discharge.

A total of 7892 patients were initiated on a PPI. Out of a mean 217 PPI days filled per patient, 194 (89%) were for the original discharge agent. During the follow-up year, 14.5% of patients filled a prescription for a PPI other than the discharge agent. At one year following discharge, 44.5% of living patients were on the original discharge agent. The total cost for filling all PPI prescriptions over the year following discharge was $ 2.48 million (Table 2). The least expensive PPI (rabeprazole, Table 3) was dispensed to 36% of patients (Figure 1). The most expensive PPI (lansoprazole) was dispensed to 39% of patients. The calculated cost savings generated by substituting all filled PPI prescriptions with the least expensive agent was $ 1.16 million (47%). If the PPI was directly funded for all the applicable hospitalised admissions, this would cost an additional $ 109 thousand.

**Table 2:** Cost of discharge prescriptions, potential Savings with inexpensive agent and cost of inpatient drug coverage for PPI, ACE inhibitors and ARBs.

	PPI*	ACE inhibitor †	ARB‡
Number of patients, n	7 892	6 802	963
Total number of “days supplied” over year following discharge, n	1 712 782	1 896 288	266 518
Cost over year following discharge, $ CAN	$ 2 475 448	$968 419	$324 568
Calculated cost if use inexpensive agent instead, $ CAN (agent)	$1 315 660 (rabeprazole)	$ 806 874 (ramipril)	$ 310 644 (candesartan)
Potential savings, $ CAN (% of real cost)	$1 159 788(47)	$161 545 (17)	$ 13 925 (4)
Total days in hospital during index admission, n	65 935	51 302	7 190
Cost of in-hospital coverage for first discharge agent, $ CAN	$ 109 099	$ 24 922	$ 8 578

* Proton pump inhibitor: omeprazole, pantoprazole, lansoprazole or rabeprazole.

† Angiotensin-converting enzyme inhibitor: ramipril, enalapril (maleate and sodium), quinapril, fosinopril, lisinopril, benazepril, perindopril, cilazapril or trandolapril.

‡ Angiotensin receptor blocker: losartan, candesartan, irbesartan, valsartan, telmisartan or eprosartan.

**Table 3:** Cost in $ CAN of one pill at equivalent doses for PPI, ACE inhibitors and ARB groups*.

	Least expensive agent, dose (cost)	Commonly prescribed agents, dose (cost)	Most expensive agent, dose (cost)
PPI†	Rabeprazole 20 mg ($0.65)	Pantoprazole 40 mg ($0.98–1.96)	Lansoprazole 30 mg ($1.0–2.0)
ACE inhibitor‡	Lisinopril 10 mg ($0.32)	Ramipril 2.5 mg ($ 0.38) Perindopril 4 mg($ 0.75–0.78)	Quinapril 10 mg ($0.85)
ARB	Candesartan 8 mg ($1.14)	Valsartan 80 mg ($1.16–1.18)	Losartan 50 mg ($1.21–1.25)

* Cost obtained from Ontario Drug Benefit formulary prices in effect from April 1^st^ 2008 to March 31st 2010 [27].

† Proton pump inhibitor: omeprazole, pantoprazole, lansoprazole or rabeprazole.

‡ Angiotensin-converting enzyme inhibitor: ramipril, enalapril (maleate and sodium), quinapril, fosinopril, lisinopril, benazepril, perindopril, cilazapril or trandolapril.

¶ Angiotensin receptor blocker: losartan, candesartan, irbesartan, valsartan, telmisartan or eprosartan.

### ACE Inhibitors

**Figure 2:**
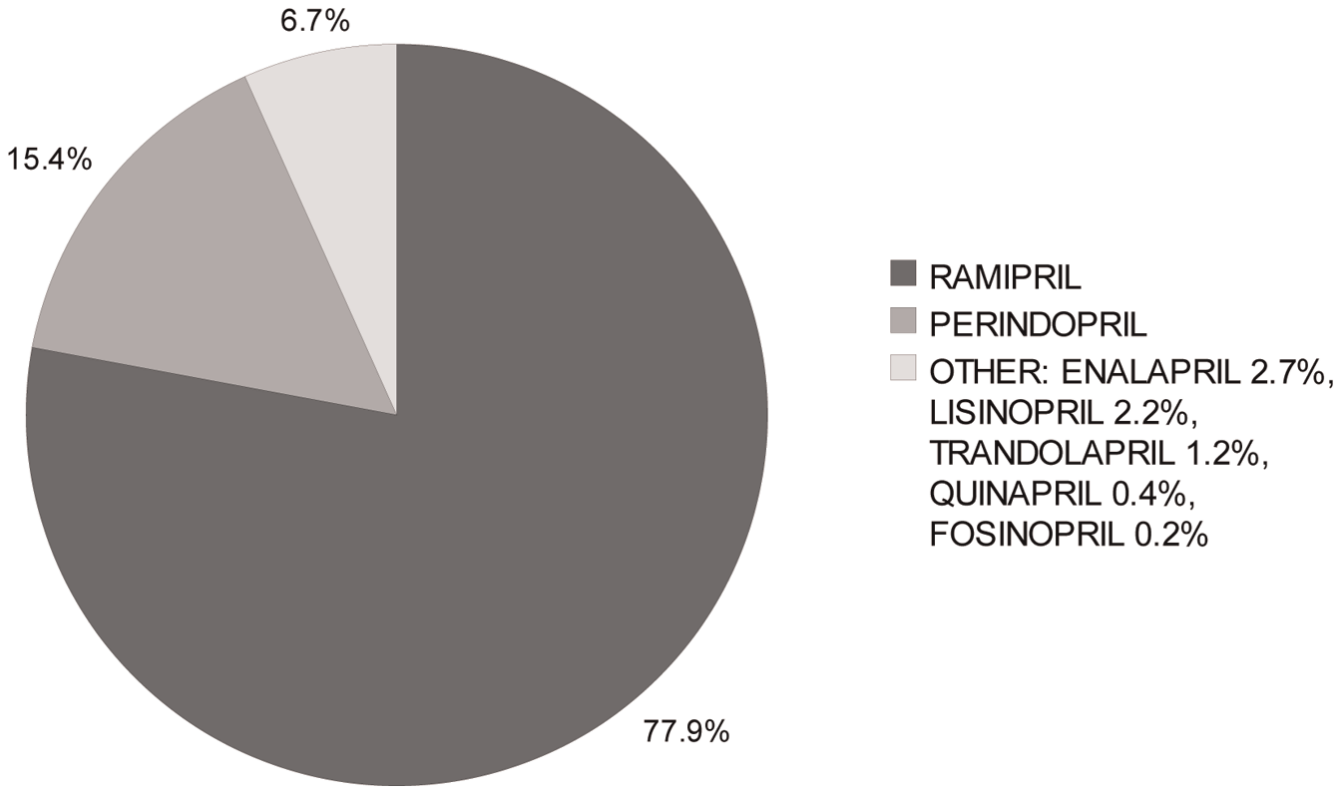
First Angiotensin-Converting Enzyme Inhibitor Prescription Filled After Hospital Discharge.

A total of 6802 patients were initiated on an ACE inhibitor. Out of a mean 278 ACE inhibitor days filled per patient, 272 (98%) were for the original discharge agent. During the follow-up year, 3.5% of patients filled a prescription for an ACE inhibitor other than the discharge agent. At one year following discharge, 59.3% of living patients were on the original discharge agent. The total cost of supplying all new ACE inhibitor prescriptions over the following year was $ 968 thousand (Table 2). The least expensive ACE inhibitors were lisinopril and ramipril. While 78% of patients were discharged on ramipril, only 2% were started on lisinopril (Figure 2). Fifteen percent of patients were discharged on perindopril, which was proprietary and one of the most expensive agents (Table 3). The calculated cost savings by substituting all filled ACE inhibitor prescriptions for lisinopril was $ 121 thousand (13%). Replacing all ACE inhibitor prescriptions with ramipril yielded cost savings of $162 thousand (17%). Supplying ACE inhibitors to these patient in hospital would cost an additional $ 25 thousand.

### Angiotensin Receptor Blockers

**Figure 3:**
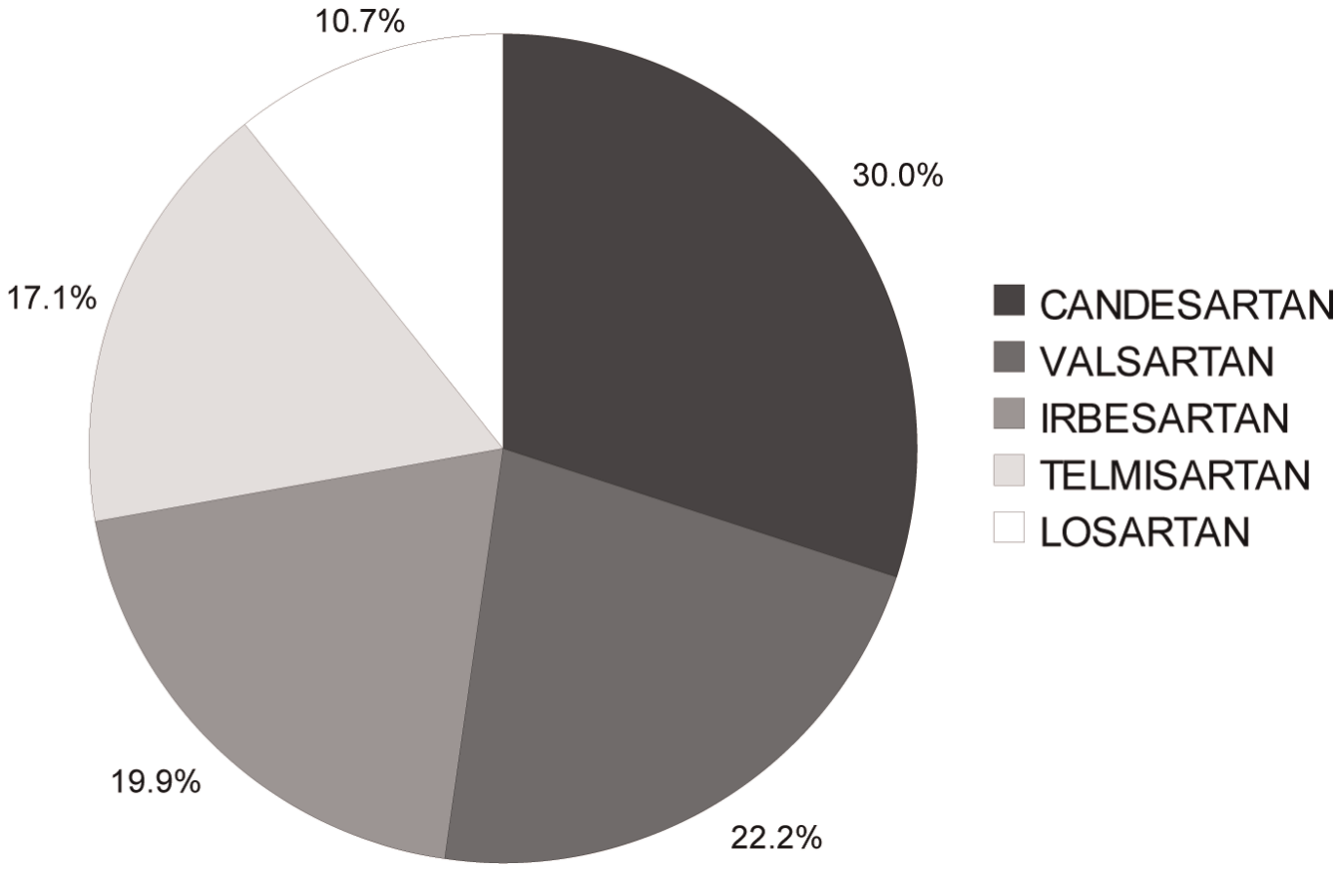
First Angiotensin Receptor Blocker Prescription Filled After Hospital Discharge.

A total 963 patients were initiated on an ARB. Out of a mean 277 ARB days filled per patient, 269 (97%) were for the original discharge agent. During the follow-up year, 4% of patients filled a prescription for an ARB other than the discharge agent. At one year following discharge, 59.5% of living patients were on the original discharge agent. The total cost for filling all ARB prescriptions over the following year was $ 325 Thousand (Table 2). Eprosartan is the least expensive agent at the standard daily dose, yet does not provide as many dose formulations as other ARBs and no patients in our cohort were discharged on this medication. Candesartan was the least expensive option with multiple dosing options (Table 3), and it represented 30% of first prescriptions (Figure 3). The most expensive agent (losartan) cost 9% more than the equivalent dose of candesartan (Table 3). Replacing all ARB prescriptions with the equivalent dose of candesartan would have saved $14 thousand (4%). Supplying ARBs to these patients in hospital would have cost an additional $ 8.5 thousand at a maximum.

## Discussion

Our study used administrative databases to determine the potential cost savings if selected drugs initiated in hospital were substituted with the least expensive agent in each class. We found that the vast majority of people continued their prescription following hospital discharge. Agent selection in some drug classes tended to favour the extremes; there were high prescription rates for both inexpensive and more expensive, proprietary agents. Our analysis showed that if the least expensive medication had been selected in hospital, $ 1.3 Million could have been saved over one year following discharge. The cost of direct inpatient drug coverage would have been less than the generated savings in all groups and less than 15% of savings for the PPI and ACE inhibitor groups.

Overall, theoretical cost-savings were modest at 35%. These were highest in the PPI group, where only 36% of patients were prescribed the least expensive agent at discharge. In our study period, this class also showed the greatest variation in unit price, as both generic and proprietary agents were simultaneously available. The extent of savings were not as great for ACE inhibitors or ARBs. This might be explained by smaller price differences between agents as well as higher market share of inexpensive agents during our study period.

Patients in our study had little incentive to select a cheaper drug. In Ontario, only higher-income patients pay a $100 annual deductible before ODB coverage occurs. Copayments to the ODB are a set per-prescription cost that does not vary depending on agent selection or drug price [22]. For this reason, prescriber preference becomes the main driver of long-term costs. Formulary harmonisation is one way to change prescriber practices to reduce long-term public drug costs.

“Harmonization” of inpatient and outpatient formularies does occur elsewhere. Some Health Maintenance Organisations in the United States combine purchasing for their hospitals and clinics in order to decrease overall acquisition costs [30]. New Zealand has also moved to a model of central contract negotiations for both inpatient and outpatient medications. While this strategy resulted in cost savings, hospital pharmacists cited loss of drug access, inferior formulary products as well as occasional drug shortages as disadvantages of this system [31].

Harmonising inpatient prescriptions with outpatient formularies may require provincially-administered therapeutic substitution policies to encourage selection of less expensive agents. Therapeutic substitution has been criticized for a few reasons. Many question the notion of a “class effect”, as data have shown within-class mortality differences [32]. This issue has been central to some well-publicized legal cases pitting governmental agencies against the pharmaceutical industry [33]. Others claim that therapeutic substitution limits physician autonomy [34]. However, opportunities for special requests can help to keep options open for prescribing physicians.

Reference-based pricing is a drug-funding policy that limits medication reimbursement to the price of the least expensive agent within a class. In British Columbia, this strategy succeeded in shifting prescription trends toward cheaper agents [35], [36]. A report from the Ministry of Health of Manitoba suggested that a reference-based pricing model would have saved Manitoba’s Pharmacare $ 2.2 Million (28%) in 2000–2001 on ACE inhibitors and ARBs alone [37]. The savings were further increased by adding mandated reduced generic pricing as occurs in several provinces [38], [39].

Provincial drug programs could also achieve savings if they implemented reference-based pricing for the drugs listed on the outpatient formulary. However, without formulary harmonisation, agent selection might be changed following hospital discharge. The advantage of harmonisation over a policy of reference-based pricing alone is that there is no need to undergo any “switching” process. Continuation requires no further cost or intervention, and as a result non-adherence may be less likely. Nonetheless, reference-based pricing has the advantage of targeting all new prescriptions rather than just those initiated in hospital.

Strengths of this study include its large sample size. Using new prescriptions filled seven days post discharge allowed us to capture the cost of medications prescribed after hospital admission only [8], [40]. By studying three different drug classes, we were better able to evaluate hospital discharge prescription practices, and to identify areas where efforts at harmonisation may be most worthwhile. Our cost calculation was done using precise data, verified against formulary prices in effect at the time. Our cost-comparison is time-specific and sensitive to cost and policy changes. For example, recent ODB pricing policy has further reduced generic drug prices to 25% of the brand price [38]. As new drugs emerge, wide price gaps between proprietary and generic agents within a class will likely be a recurring phenomenon. While the degree of cost-savings generated by a harmonisation policy may vary by drug class, the concept remains valid for all price points. Furthermore, these medications are considered to be chronic therapies. As such, savings would be ongoing for many years.

### Limitations

While the harmonisation model rests on the inpatient formulary, our study is limited by the fact that we only have data on prescriptions filled by outpatients. We were not able to ascertain inpatient drug therapy using the administrative data available to us, and used prescriptions filled 7 days post-discharge as a marker of inpatient medication selection. Using such a close temporal association reduces the likelihood of contamination by outpatient prescribers, however this cannot be entirely ruled out.

Furthermore, our estimate of cost-savings in the harmonisation model may be overestimated. A small number of patients who changed agents during the year of follow-up would presumably escape the influence of the inpatient formulary on long-term therapy. This effect may be greatest in the PPI group, where 11% of PPI days were filled with a non-discharge PPI.

Our ability to predict cost-savings is also limited by our lack of knowledge about inpatient medication costs. We do not know if local negotiations result in lower prices than those quoted by the ODB, because information about supplier contracts with hospitals is confidential [41]. While a harmonisation policy could save money on certain medications, it is possible that costs for other (eg: intravenous or uninsured) medications would increase as a result of lost contracts or bundling agreements with pharmaceutical suppliers. Nonetheless, the ODB possibly obtains more favorable pricing on most medications, given that it has more purchasing power.

### Conclusion

For selected chronic disease medications, we found that drug selection in hospital was strongly associated with long-term outpatient prescribing. Yet it was not reflective of outpatient prices. The least expensive agent in the class was seldom chosen. Our results show that this can result in incremental outpatient costs. In-hospital selection of agents with the lowest outpatient prices is one way to limit the long-term costs of chronic drug therapy. In an era of rising healthcare costs a harmonised approach makes economic sense.

## Supporting Information

Table S1 **Dose Equivalencies for Proton-Pump Inhibitors.** Doses have been adapted to reflect available dose formulations from the World Health Organisation’s Defined Daily Doses [28].
(DOC)

Table S2 **Dose Equivalencies for Angiotensin-Converting Enzyme Inhibitors.** Doses have been adapted to reflect available dose formulations from the World Health Organisation’s Defined Daily Doses [28].
(DOC)

Table S3 **Dose Equivalencies for Angiotensin Receptor Blockers.** Doses have been adapted to reflect available dose formulations from the World Health Organisation’s Defined Daily Doses [28].
(DOC)
