# Supplementary material for: Potential Savings of Harmonising Hospital and Community Formularies for Chronic Disease Medications Initiated in Hospital
Source: PLoS One. 2012 Jun 26;7(6):e39737. doi: 10.1371/journal.pone.0039737 (PMC3383681; doi:10.1371/journal.pone.0039737)
Supplement: Table S2 — Dose Equivalencies for Angiotensin-Converting Enzyme Inhibitors. Doses have been adapted to reflect available dose formulations from the World Health Organisation’s Defined Daily Doses [28]. (DOC) [file pone.0039737.s002.doc]

| Drug Name | Equivalent Dose |
| --- | --- |
| Ramipril | 2.5 mg |
| Enalapril maleate | 10 mg |
| Enalapril sodium | 8 mg |
| Quinapril | 10 mg |
| Fosinopril | 10 mg |
| Lisinopril | 10 mg |
| Benazepril | 10 mg |
| Perindopril | 4 mg |
| Cilazapril | 2.5 mg |
| Trandolapril | 2 mg |
