# Supplementary material for: Potential Savings of Harmonising Hospital and Community Formularies for Chronic Disease Medications Initiated in Hospital
Source: PLoS One. 2012 Jun 26;7(6):e39737. doi: 10.1371/journal.pone.0039737 (PMC3383681; doi:10.1371/journal.pone.0039737)
Supplement: Table S3 — Dose Equivalencies for Angiotensin Receptor Blockers. Doses have been adapted to reflect available dose formulations from the World Health Organisation’s Defined Daily Doses [28]. (DOC) [file pone.0039737.s003.doc]

| Drug Name | Equivalent Dose |
| --- | --- |
| Losartan | 50 mg |
| Candesartan | 8 mg |
| Irbesartan | 150 mg |
| Valsartan | 80 mg |
| Telmisartan | 40 mg |
| Eprosartan | 600 mg |
